# The Influence of Anaesthetic Drugs on the Laryngeal Motion in Dogs: A Systematic Review

**DOI:** 10.3390/ani10030530

**Published:** 2020-03-22

**Authors:** Elisabeth Ranninger, Marta Kantyka, Rima Nadine Bektas

**Affiliations:** 1 Department of Clinical Diagnostics and Services, Section of Anaesthesiology, Vetsuisse Faculty University of Zurich, Winterthurerstrasse 260, 8057 Zurich, Switzerland; 2 Department of Clinical Veterinary Medicine, Section of Anaesthesiology, Vetsuisse Faculty University of Bern, Hochschulstrasse 6, 3012 Bern, Switzerland

**Keywords:** laryngeal motion, anaesthesia, dogs, doxapram

## Abstract

**Simple Summary:**

Laryngeal paralysis is secondary to a loss of normal function of the larynx. Older dogs are particularly affected, with normal breathing becoming difficult. A successful diagnosis typically relies on the visualisation of either, complete, or partially absent, laryngeal movements. The use of anaesthesia drugs to provide sedation and stress relief is most commonly necessary during the diagnosis of laryngeal paralysis. While, the excessive administration of anaesthesia drugs may result in absent movements, the ideal anaesthesia regime remains unknown, and the use of sedation is questionable, given the potential for absent laryngeal movements, even in healthy dogs. In this systematic review, we found a potential benefit from using sedation during the evaluation of laryngeal function when compared to injectable anaesthetics only. The respiratory stimulant doxapram was effective in differentiating normal dogs from dogs with laryngeal paralysis but has associated safety hazards.

**Abstract:**

Anaesthetic drugs are commonly used during the evaluation of laryngeal function in dogs. The aim of this review was to systematically analyse the literature describing the effects of anaesthetic drugs and doxapram on laryngeal motion in dogs and to determine which drug regime provides the best conditions for laryngeal examination. PubMed, Google Scholar, and EMBASE databases were used for the literature search up to November 2019. Relevant search terms included laryngeal motion, anaesthetic drugs and dogs. Studies were scored based on their level of evidence (LoE), according to the Oxford Centre for Evidence-based Medicine, and the quality was assessed using the risk-of-bias tool and SIGN-checklist. In healthy dogs, premedication before laryngeal examination provided better examination conditions and maintained overall adequate laryngeal motion in 83% of the studies. No difference in laryngeal motion between induction drugs was found in 73% of the studies but the effects in dogs with laryngeal paralysis remain largely unknown. Doxapram increased laryngeal motion in healthy dogs without serious side effects, but intubation was necessary for some dogs with laryngeal paralysis. Methodological characteristics varied considerably between studies, including the technique and timing of evaluation, number of assessors, study design, drug dose, combinations, route and speed of administration.

## 1. Introduction

The use of anaesthetic drugs to provide a light plane of anaesthesia is usually inevitable in dogs, to allow visualisation of arytenoid motion during the diagnosis of laryngeal paralysis. In healthy dogs, bilateral arytenoid abduction is normally observed during inspiration. However, if the anaesthetic depth is excessive, arytenoid function can be lost. Most anaesthetics inhibit arytenoid motion in a dose-depended manner, possibly leading to a false diagnosis of laryngeal paralysis [1]. 

Laryngeal paralysis has a high prevalence in canines, in which both the hereditary or acquired form exist [2]. The most common acquired form is idiopathic, secondary to a progressive degenerative neuropathy, and while this can occur in all breeds, it is typically found in older-aged dogs [3]. The diagnosis of laryngeal paralysis is typically made either via direct observation or video laryngoscopy. However, no standardisation for this procedure has been described [4,5]. The ideal anaesthetic protocol for examination should provide adequate sedation and jaw relaxation, without decreasing arytenoid motion and causing minimal respiratory depression. Providing an ideal anaesthetic plane can be challenging, as slow titration of anaesthetic drugs can result in excitement, whereas fast titration might ensure absent reflexes. It might also result in the suppression of arytenoid motion and subsequent intubation of the trachea. The majority of studies in veterinary medicine use different anaesthetic protocols during the diagnosis of laryngeal paralysis and different evaluation methods. While some authors avoid the use of sedatives [6], others recommend their use to provide better conditions for examination, in adjunct to doxapram to stimulate laryngeal motion [7]. To date, there is no consensus over either, sedative or anaesthetic management, or for the use of respiratory stimulants during the evaluation of the laryngeal function in dogs.

Therefore, the aims of this review were: (i) To determine the extent to which premedication drugs are used during the diagnosis of laryngeal paralysis in dogs and to determine their effects on laryngeal motion; (ii) to determine the effects of induction agents on laryngeal motion and the diagnosis of laryngeal paralysis; (iii) to establish whether any drug regime provides the best conditions and improved safety for the dogs during laryngeal examination; and (iv) to establish whether the use of doxapram is indicated and safe for the diagnosis of laryngeal paralysis. 

## 2. Materials and Methods 

**Figure 1:**
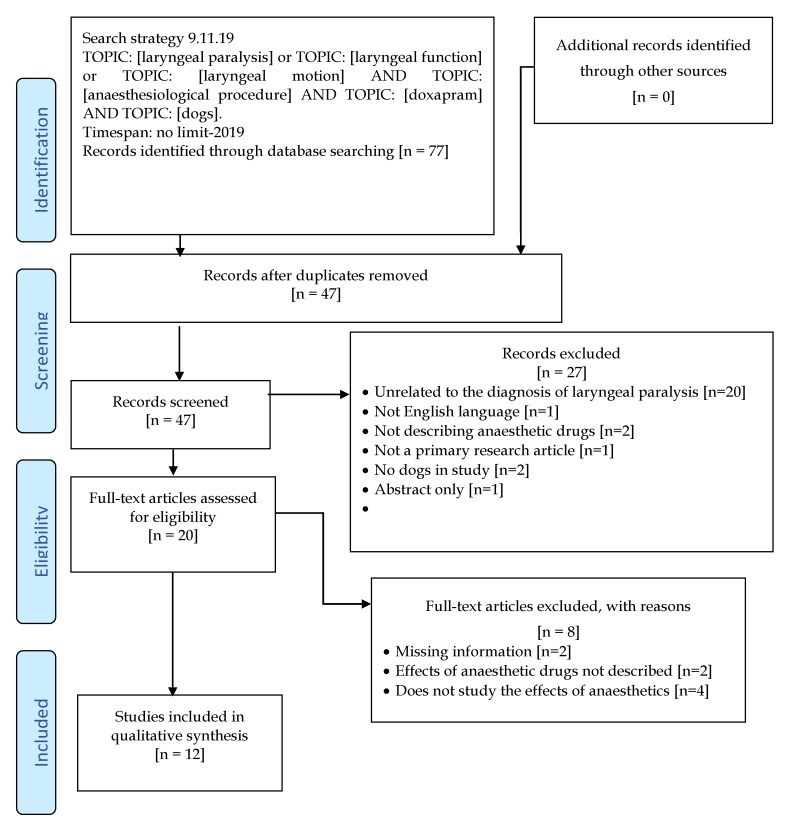
The review structure and search strategies evaluating the effects of anaesthetic drugs on the laryngeal motion in dogs [9].

The Preferred Reporting Items for Systematic Reviews and Meta-analyses (PRISMA) guidelines and checklist were consulted and adhered to during the review design and development phase [8]. Three internet databases were searched to identify the studies for review, including PubMed, Google Scholar, and the EMBASE database. The full search strategy is shown in Figure 1. Papers available through the University of Zurich were also used. Keywords entered were “laryngeal paralysis”, “laryngeal function”, “laryngeal motion”, “anaesthetic drugs”, “anaesthesiological procedure”, “dogs” and “doxapram”. Articles were excluded if they met any of the following exclusion criteria: (i) Did not describe the anaesthetic regime; (ii) belonged to a different species other than dogs; (iii) failed to report relevant anaesthetic drug information such as resulting effect, dosage or route of administration. In the first stage, the effects of premedication drugs on the laryngeal motion in dogs were evaluated. In the second stage, induction drugs were assessed. Only primary research articles in English, describing the use of anaesthetic drugs (including the dose and route of administration) during the evaluation of laryngeal function in dogs were selected (Table 1). The quality and method of laryngeal examination were compared between trials. In the third stage, the use of doxapram for the diagnosis of laryngeal paralysis was evaluated. Articles were categorised on the basis of premedication, induction and stimulating agent used (Table 2, Table 3 and Table 4). 

### Assessment of Study Quality and Level of Evidence 

Manuscripts were scored based on their level of evidence (LoE) following the 2011 Oxford Centre for Evidence-based Medicine approach, which includes high quality levels (I-LoE) with evidence obtained from a systematic review and lower quality (II-LoE) with evidence obtained from properly designed, randomised controlled trials or non-randomised trials (III-LoE) [20]. The methodological quality of the studies was assessed according to the modified SIGN Methodology Checklist 2 of randomised controlled trials (RCTs), in which a bias rating of low (++) moderate (+), or high (-) is provided, based on ten questions [21]. The modified checklist consisted of 10 criteria, with each criterion receiving a score of either one or two points. When all criteria were adequately described and the sum of the scores resulted in 10–11 points, a double plus was assigned. A plus sign was given to studies scoring over seven points and a minus for studies scoring under seven points. Data extraction and risk-of-bias assessment were performed by two individual evaluators, with a third involved in cases of disagreement. Table 5 shows the modified methodology checklist, including the scoring system, and LoE.

## 3. Results

The first literature search identified 47 potentially eligible articles, of which 27 articles were excluded after reviewing the abstracts, as they did not meet the inclusion criteria or met the exclusion criteria. Of the 20 studies included for full-text analysis, eight were excluded by the exclusion criteria. Twelve prospective studies were included in the qualitative synthesis, including eight randomised studies, two controlled, and one clinical study (Table 1). The total number of dogs involved in the remaining 12 reviewed articles were 232, of which 54 dogs were research animals. Three studies failed to report the source of the dogs. The sample sizes varied considerably, with a mean of 19 (range 6–48) dogs per study. Three studies reported a prospective power calculation [13,14]. Most studies (83%) used a randomised method of drug allocation but not all described an acceptable method of randomisation (Table 5). Some studies (66%) were designed in a crossover fashion and the evaluators were unaware of the treatment allocation. Only one of the studies included dogs with suspected laryngeal paralysis, in a non-blinded, non-randomised design [17]. Most studies (83%) provided a direct comparison between anaesthetic drugs, with two studies including a control group [10,13]. A total of 58% of studies evaluated laryngeal function objectively by measurement of the normalised glottal gap area (NGAA) or normalised rima glottides surface area (RGSA) via video laryngoscopy. Few studies (33%) used a subjective scoring method via direct visualisation. Methodological characteristics of the studies are summarised in Table 1. In Table 5, the level of evidence (LoE) and the quality assessments are shown. 

### 3.1. Effects of Premedication Drugs and Influence on the Quality of Laryngeal Examination

Premedication was used in 66% of the studies during the evaluation of laryngeal motion. Five out of 8 (62%) studies did not report the conditions or quality of sedation during the laryngeal examination. In the remaining studies, good evaluation conditions were found in premedicated dogs (Table 2). In two comparative studies, the direct effects between premedication and lack of premedication on the quality of laryngeal examination were analysed. A significant improvement in laryngeal exposure scores was obtained in dogs premedicated with butorphanol and acepromazine when compared to non-premedicated dogs [7,13].

In the first stage, studies (level II) were evaluated for the use of premedication drugs during the evaluation of laryngeal motion in dogs (Table 2). Premedication drugs were used in 8 out of 12 (66%) studies. Of these, 25% used one sedative only, whereas 75% reported using at least two sedatives. A total of six premedication drugs were evaluated, including four opioids (butorphanol, methadone, hydromorphone and oxymorphone), one alpha-two adrenergic agonist (dexmedetomidine) and one phenothiazine derivate (acepromazine). The effects of premedication drugs were evaluated after the induction of anaesthesia in all studies except for one [7]. In this study, the effects of premedication were studied independently of induction drugs, concluding that laryngeal function was present in all sedated dogs, but was absent when propofol only was administered. By contrast, one level II study providing a direct comparison between the effects of premedication and lack of premedication concluded that, while premedication improved the quality of the laryngeal examination, 50% of premedicated animals had absent laryngeal function [13]. Two studies using premedication failed to analyse the effects of premedication on laryngeal function [16,19]. The overall laryngeal function of the studies analysing the effects of premedication concluded that premedication maintained (75% or higher) adequate laryngeal motion in 5 out of 6 (83%) of healthy dogs and provided better conditions for the laryngeal examination.

#### 3.1.1. Dexmedetomidine

The effects of dexmedetomidine were evaluated in one study, which was the only titrating premedication to effect (Table 2). In this study, Degroot et al. determined the ability to evaluate laryngeal function under sedation with dexmedetomidine alone, or in combination with butorphanol, hydromorphone or compared to propofol alone [7]. Drug dosages can be found in Table 2. Laryngeal function was observed in all dogs receiving dexmedetomidine, with, or without, opioids but was absent in two dogs in the propofol group. It was concluded, that dexmedetomidine sedation does not inhibit normal laryngeal motion and provided good conditions for the laryngeal examination, even when combined with opioids. One dog vomited after dexmedetomidine, but no other side effects were reported. No dog required intubation at the maximum dose of 15 µg kg^−1^ intravenous (IV). Interestingly, laryngeal motion after the administration of doxapram was greater for dogs receiving dexmedetomidine alone, suggesting that dexmedetomidine had the least depressive effect on laryngeal motion.

#### 3.1.2. Butorphanol 

The effects of either butorphanol, methadone or hydromorphone on laryngeal function were evaluated in all studies involving premedication (Table 2). Dosages for IV or intramuscular (IM) butorphanol ranged between 0.2 to 0.5 mg kg^−1^. The effects of different dose range were not analysed. Butorphanol was used in combination with another premedication drug in all studies, except for two, in which butorphanol was the sole premedication drug [16,20]. Arytenoid motion was graded subjectively by a blinded assessor in a crossover study and was found present in all healthy dogs premedicated with butorphanol (0.5 mg kg^−1^ IV) when administered 5 min prior to induction of anaesthesia with thiopental or propofol [18]. The effects of butorphanol were not directly analysed or compared with a control group (placebo). 

#### 3.1.3. Acepromazine and Butorphanol

Acepromazine was combined with butorphanol for premedication in 50% of the studies. Dosages for acepromazine ranged from 0.02 to 0.2 mg kg^−1^ (Table 2) and were administered either IV, IM or subcutaneously (SQ), either 5 or 20 minutes before induction of anaesthesia. Larger doses of acepromazine combined with butorphanol did not appear to affect laryngeal motion in healthy dogs when anaesthesia was induced with isoflurane in a LoE III [17] and LoE II study [6]. In contrast, when acepromazine and butorphanol were used for premedication, a lack of arytenoid motion was observed in 50% of the dogs induced with either propofol or alfaxalone in a controlled randomised trial (level II) [13]. 

#### 3.1.4. Acepromazine and Thiopental, Propofol, Isoflurane

While in healthy dogs premedicated with acepromazine, the overall dose of the induction agent propofol or thiopental was reduced, the laryngeal motion was absent in some dogs or significantly decreased when compared to examinations with thiopental alone or acepromazine–butorphanol and isoflurane combinations [6].

#### 3.1.5. Methadone, Hydromorphone, Oxymorphone

The use of methadone, hydromorphone or oxymorphone was reported in 3 studies [6,7,17] (Table 2), however one failed to report the degree of laryngeal motion in dogs premedicated with oxymorphone [6]. IM premedication with methadone (combined with acepromazine) 30 min prior to laryngeal examination resulted in a 15% loss of detectable arytenoid motion when propofol or alfaxalone were used as induction agents [12]. In contrast, when hydromorphone was combined with dexmedetomidine, titrated to effect, no detrimental effects were evidenced on the laryngeal motion and sedation was excellent to perform a laryngeal examination [7]. 

### 3.2. Effects of Induction Drugs and Influence on the Quality of Laryngeal Examination

The second stage evaluated the effects of induction drugs on laryngeal function in dogs (Table 3). A total of 33% of the studies used induction agents only. A total of five induction drugs were evaluated, including alfaxalone, propofol, isoflurane, methohexital and thiopental. Adjuvant drugs were used in 41% of the studies and included ketamine and diazepam. Propofol was used in each study, whereas alfaxalone and thiopental were used in 41%, and 33% of the studies, respectively. No difference in laryngeal motion between induction drugs [when assessed shortly after the induction of anaesthesia] was found in 73% of the studies [6,13,14,17,18]. In the four studies in which laryngeal function was found to be decreased, an objective scoring method had been used in three studies (LoE II) [6,7,13], and a subjective score in one study [11]. Of all studies, only one study compared the effects of induction drugs in both, premedicated and non-premedicated dogs, concluding that mainly non-premedicated dogs had absent laryngeal function regardless of the induction agent [13]. 

When premedication was omitted, administration of alfaxalone or propofol alone did not provide a good quality of examination when assessed by blinded observers in a controlled crossover study [13]. Also, in a crossover study, administration of propofol only resulted in insufficient sedation, gagging, paradoxical laryngeal movements or transient paralysis [7]. In contrast, when thiopental was used alone, better conditions for the oral laryngeal examination were achieved [15]. In dogs anaesthetised with ketamine-diazepam, one study found poorer examination conditions [18], and another found no differences [16]; however, in that study the agreement between assessors was poor.

#### 3.2.1. Propofol

In six studies, no statistically significant differences were found in laryngeal function after the administration to effect of propofol (5.1 mg kg^−1^ IV; range 3.6–6.8 mg kg^−1^ IV) when compared to alfaxalone, methohexital, thiopental, and ketamine-diazepam mixture. Also, when propofol was combined with diazepam, no significant differences in laryngeal motion were found when compared to alfaxalone or thiopental [14]. Smalle et al. concluded, by direct observation, that propofol resulted in shorter examination times and better laryngeal abduction scores when compared with alfaxalone and thiopental [15].

In contrast, two studies showed significantly poorer laryngeal function after the induction of anaesthesia with propofol (mean dose 6.35 mg kg^−1^ IV) when administered to effect in non-premedicated dogs in studies with objective scoring methods [7,13]. While the difference was recorded in only two dogs in one study, both dogs were false-positive for laryngeal paralysis [7]. Overall, the effects of propofol were found to vary with individual studies. While some authors reported no respiratory depression, others found propofol to cause apnoea [14,16,18] or absent laryngeal movements [6].

#### 3.2.2. Alfaxalone

No significant differences were found in laryngeal function after alfaxalone (2.6 mg kg^−1^ IV; range 2–3.2 mg kg^−1^ IV) (Table 3) administration in 3 studies, when directly compared to propofol and thiopental in premedicated [12] and non-premedicated dogs [12,14,15]. In contrast, alfaxalone administered at a lower dose (1.9 mg kg^−1^ IV; range 1.5–2.3 mg kg^−1^ IV), did not preserve the arytenoid function when compared to propofol [13,16]. None of the studies combined alfaxalone with an adjuvant drug. In the studies that found alfaxalone comparable to propofol, one used premedication and most used a subjective scoring system (Table 1). 

#### 3.2.3. Thiopental

Thiopental was used as an induction agent in four randomised level II studies, in which a total of 28 dogs were enrolled in a crossover design. Arytenoid motion was graded by blinded observers (Table 1) objectively in two of the studies [6,14] and subjectively in another two [15,18]. In all studies, no statistically significant differences were found in laryngeal function after the induction of anaesthesia with thiopental (14.1 mg kg^−1^ IV; range 10.4–17.8 mg kg^−1^ IV) when compared to alfaxalone, propofol, propofol-diazepam or ketamine-diazepam. However, significantly greater laryngeal motion was found in dogs recovering from anaesthesia after previous induction with thiopental (14 mg kg^−1^ IV) [6].

#### 3.2.4. Ketamine-Diazepam

Absent arytenoid movements and lower laryngeal exposure scores due to excessive jaw tone or laryngospasm were reported in two blinded, crossover studies using ketamine-diazepam [6,18]. Gross et al. administered ketamine-diazepam over 1 minute to effect, whereas Jackson et al. did not report the speed of administration [6]. When ketamine (2 mg kg^−1^ IV) was combined with propofol (2.4 mg kg^−1.^ IV), a tendency to respiratory depression, apnoea and low pulse oximetry values were found [16].

#### 3.2.5. Methohexital

Tachycardia, seizure-like activity, vomiting and regurgitation were reported in 25% of the dogs in a study evaluating methohexital for its effects on laryngeal motion [10]. No difference between methohexital and propofol was detected regarding laryngeal function.

#### 3.2.6. Isoflurane Mask Induction

Arytenoid motion assessed by an objective scoring method was preserved with isoflurane anaesthesia [mask induction] in two prospective studies in premedicated dogs [6,17]. In one of the studies, neither the isoflurane concentration during induction of anaesthesia nor the quality of induction of anaesthesia were reported [6]. However, it was reported that high acepromazine dosages (0.2 mg kg^−1^ IM) were necessary to allow mask induction. Laryngeal motion was maintained adequately in a non-blinded level III study, when a face mask delivering an isoflurane concentration of 3–5%, until sufficient jaw relaxation, was achieved [17].

### 3.3. Agreement Between Laryngeal Function Assessors

The agreement between assessors of laryngeal function was excellent in only one study [12]. Brown et al. found disagreements in the assessment of laryngeal function, swallowing and the incidence of laryngospasm between direct and masked observers [10]. Also, disagreement between observers was reported by McKeirnan et al. in 58% of the cases, with some dogs being classified by some observers as having laryngeal paralysis and healthy by others [16]. 

### 3.4. Respiratory Stimulants

#### 3.4.1. Doxapram

Doxapram was evaluated as a respiratory stimulant in 67% of the studies and successfully increased laryngeal motion in 75% of these studies (Table 4). In a crossover, blinded level II study, doxapram improved laryngeal function when assessed objectively only in dogs receiving dexmedetomidine alone and not when combined with opioids or propofol [7]. In the only controlled study, doxapram resulted in no improved laryngeal motion in all non-premedicated dogs, however the agreement between assessors was inconsistent [10]. Doxapram was effective in stimulating laryngeal function in non-premedicated dogs [11]. The remaining six studies evaluated doxapram in premedicated dogs. In one of these studies, laryngeal function was successfully observed in 40 premedicated dogs with previously lacking arytenoid motion in a crossover design (level II) [13]. In that study, doxapram was administered at the lowest described dose of 0.25 mg kg^−1^ IV, however improved laryngeal function was not obtained in 50% of the dogs in the alfaxalone group. Overall described dosages ranged from 0.25 mg kg^−1^ IV to 2.5 mg kg^−1^ IV (Table 4). In dogs with laryngeal paralysis, the administration of doxapram resulted in passive or paradoxical movements and intubation was necessary [17]. In healthy dogs, side effects were limited to awakening, excitement and increased respiratory drive (Table 4). 

#### 3.4.2. Mechanical Stimulation

Mechanical stimulation of the larynx with a cotton bud was less effective in stimulating laryngeal function than doxapram in dogs [11]. 

## 4. Discussion

Over the past 17 years, five premedication drugs and eight induction drugs were evaluated for their effects on the arytenoid motion in dogs. The evidence from the studies, included in this review, demonstrated variably different effects of anaesthetic drugs on the laryngeal motion in dogs. 

Overall, most studies concluded no significant differences in laryngeal motion between sedatives, including butorphanol, acepromazine, dexmedetomidine, and between the induction drugs alfaxalone, propofol or thiopental. However, 96% of analysed subjects in the included studies were healthy dogs. It is possible, that the effects of anaesthesia drugs on laryngeal motion are more pronounced in dogs with laryngeal paralysis, due to an underlying neuropathy of the recurrent laryngeal nerve, innervating the crycoarytenoid dorsalis muscles. Therefore, in dogs with some degree of laryngeal paralysis, possible upper airway obstruction and life-threatening respiratory distress [17] ought to be considered, regardless of the anaesthetic protocol used. 

In this review, most studies (83.3%) reported no statistically significant reduction in laryngeal motion in premedicated dogs, with the majority concluding that laryngeal motion is maintained despite the use of premedication. A low-risk-of bias, LoE II study concluded that laryngeal motion remains above 75% despite acepromazine and methadone premedication [12]. Nevertheless, one study found a statistically significant decrease in laryngeal function during the recovery phase in premedicated dogs, when compared to non-premedicated dogs [6]. However, this study used the highest dose of acepromazine (0.05 mg kg^−1^ IM) among all studies using later injectable induction agent, a fact which might account for their results. In general, the effects of acepromazine on laryngeal motion are controversial. Relatively low doses, combined with an injectional induction (alfaxalone or propofol), resulted in absent laryngeal motion, whereas significantly higher doses of acepromazine (0.2 mg kg^−1^ IM), followed by inhalational anaesthesia (isoflurane), maintained laryngeal motion [6,17]. Based on these results, isoflurane mask induction might, perhaps, be considered for the evaluation of laryngeal function. Nevertheless, a possible risk of bias must be considered, given that the study by Tobias et al. was classified as a LoE III and was non-randomised and non-blinded [17]. Still, possible benefits from inhalational mask induction are usually lost, due to inhalant induction being more likely associated with complications (movement, stress and the need for restraining due to the pungent and irritating smell, as well as potential health hazards to the personnel from repeated exposure to volatile anaesthetics) [22,23,24]. Among the studies evaluating the effects of premedication on laryngeal function, Radkey et al. contrasts positively from the others [13]. Firstly, their study design is based on an objective assessment in a crossover design with a saline control group, which no other study included, when analysing premedication drugs. Secondly, the examination conditions during the laryngeal examinations were adequately reported and drug choice, as well as drug dosages and administration rate may reflect daily clinical conditions. Their results are intriguing, since premedicated dogs had a higher degree of laryngeal function (50%) after induction of anaesthesia than non-premedicated dogs (0–20%), who required a significantly higher induction dose. Furthermore, after administration of doxapram, the increase in laryngeal function was higher in premedicated dogs than non-premedicated dogs, suggesting that the incidence of false-positive diagnosis is higher in non-premedicated dogs. A possible explanation lies in the fact that premedicated dogs required fewer induction drugs, and therefore, the drug-induced inhibition of the larynx may diminish, which has also been proposed by Degroot et al. [7]. In their study, preserved laryngeal function was found in all premedicated dogs but was absent in the same dogs anaesthetised with propofol only. This study was the only randomised crossover study to titrate induction agents, in order to effect and adds to the evidence suggesting that premedication preserves laryngeal function, possibly, to a better extent than using solely induction drugs [7,12,17,18].

The majority of studies concluded no significant differences in laryngeal motion after the induction of anaesthesia, regardless of the induction drug choice [6,10,12,14,17,18]. The same conclusion was also reached by the two studies with the lowest risk of bias [10,12]. Nevertheless, the results are somewhat conflicting. One study reported superior laryngeal motion with thiopental during the recovery phase [6]. Also, another study reported no motion in all dogs anaesthetised with alfaxalone and 20% motion in dogs anaesthetised with propofol [13], whereas another reported less motion after propofol, when compared to dexmedetomidine only [7]. 

A higher and clearer agreement seems to be found regarding the effectiveness of doxapram. The majority of studies concluded that doxapram was useful for differentiating non-affected dogs from dogs with laryngeal paralysis, with no serious side effects reported in healthy patients. Importantly, doxapram administration is associated with decreases in cerebral blood flow and increases in cerebral oxygen consumption and requirement, as well as hypertension, cardiac arrhythmias, seizures or muscle rigidity, occurring more likely with higher dosages [25,26,27,28,29,30]. Therefore, the benefits of using doxapram might not outweigh the risks. While all the studies administered doxapram as a bolus, constant rate infusion (CRI) might offer the possibility to titrate doxapram to effect and may reduce the likelihood of adverse events [31]. Given the occurrence of paradoxical motion and increased airway resistance in dogs with laryngeal paralysis, the means for quick intubation ought to be readily available when doxapram is administered [17,32].

A number of limitations and possible confounding factors must be considered in the present review. Cautious interpretation of the results is warranted because of the small number of available controlled studies and limited number of reviewed articles, with most studies, including few animals. The introduction of a statistical type-two error is particularly high in studies which include a small number of subjects, although most studies (58%) attempted to reduce this limitation with a crossover design. Additionally, the study design was not ideal in most studies, with only two including a control group [10,13] and some lacking masked observers [17,19]. Another limitation is that studies evaluated laryngeal motion at different time points after induction of anaesthesia. Labuscagne et al. recommended evaluating function after 2 minutes of induction because, in that time period, a higher number of vital breaths and laryngeal movements were observed, whereas the quality of examination was good. If the examination is either, too early or too late within the recovery phase, either absent motions or movement artefact might be present. Another limitation is that the studies did not use a consistent methodology, which undoubtedly can lead to assessment variations. Even with the use of objective scoring methods, such as the calculation of the normalised glottal gap area (NGAA) from digitised images, variations in the distance between the video scope and the glottal gap area, as well as assessments by different evaluators, can lead to a risk of bias. Also, video laryngoscopy does not necessarily correlate with clinical signs [2]. A potential source of error may be introduced, particularly when the respiratory phase is not taken into account when assessing paradoxical laryngeal motion, as paralysed arytenoid cartilages can passively move during exhalation, mimicking normal laryngeal movements [3,33].

In comparison, direct observers have no option to pause the evaluation, which could influence the ability to determine laryngeal function, especially during a fast breathing pattern. In general, subjective scoring methods have the potential to introduce individual variance and inter-individual bias when multiple evaluators performed assessments at different time points. Finally, the rate of drug administration was inconsistent between studies. All authors administered drugs to effect, the time period over which the drugs were administered was carried out mainly over one minute, so that the onset of action of some drugs might have taken longer than the waited time. Also, some drugs such as alfaxalone, induced excitement when administered slowly [13] and were therefore administered relatively fast, which may have led to lower laryngeal motion due to higher overall administered dose. Finally, the fact that studies analysed drugs in healthy animals is important to consider because their side-effects can be significantly more pronounced in older animals, where laryngeal paralysis is most frequent [3,34].

## 5. Conclusions

The goal of anaesthesia during the evaluation of laryngeal function in dogs should be aimed at providing adequate sedation, while maintaining laryngeal motion and reflexes mostly intact. While there might not be sufficient evidence to clearly recommend one single anaesthetic regime for the evaluation of laryngeal function in dogs, current evidence suggests that premedication maintains overall adequate laryngeal motion in dogs and provides better conditions for laryngeal examination than achieved with solely use of induction agents. Doxapram is effective in differentiating normal dogs from dogs with laryngeal paralysis, but has associated safety hazards.

## Figures and Tables

**Table 1:** Results and study features of standardised methodological assessment of the 12 included studies evaluating the effects of anaesthetic drugs on the laryngeal motion in dogs.

Reference	Journal	Study design	Number of Dogs	Health Status	Group Size	Prospective Power Calculation	Evaluation Method	Assessment of Laryngeal Function	Laryngeal Function Assessed during Inspiratory Cycle	Statistical Analysis
Brown et al. (2019) [10]	*Veterinary surgery*	Prospective, controlled randomised blinded	40 shelter dogs	Healthy	10/10/10/10	Yes	Normalised glottal gap area (NGAA)	Direct visualisation and video from videolaryngoscopy	Yes	ANOVA
DeGroot et al. (2019) [7]	*Veterinary surgery*	Prospective randomised crossover	8 research dogs	Healthy	8/8/8/8	No	Video laryngoscopy, normalised glottal gap area (NGAA)	Still images from videolaryngoscopy	Yes	ANOVA
		blinded								
Labuscagne et al. (2019) [11]	*Veterinary anaesthesia and analgesia*	Prospective randomized crossover	8 research dogs	Healthy	8/8/8/8/8/8	No	Visual subjective	Subjective laryngeal exposure score	Yes	Friedman rank sum test/Wilcoxon rank sum test/ANOVA/
		blinded								
Norgate et al. (2018) [12]	*Veterinary anaesthesia and analgesia*	Prospective randomized, blinded	48 client-owned dogs	Healthy brachy-cephalic	24/24	No	Video laryngoscopy, visual subjective	Subjective laryngeal exposure score and video from videolaryngoscopy	Yes	Shapiro-Wilk test/
										Chi square and Fisher’s exact tests
Radkey et al. (2018) [13]	*Veterinary anaesthesia and analgesia*	Prospective randomized controlled crossover, blinded	10 research dogs	Healthy	10/10/10/10	Yes	Normalised rima glottides surface area (RGSA)	Video and still images from videolaryngoscopy	Yes	Shapiro-Wilk test/ANOVA/Kruskal-Wallis
Ambros et al. (2018) [14]	*Canadian veterinary journal*	Prospective, crossover randomised blinded	8 client-owned dogs	Healthy	2008/8/8	Yes	Normalised glottal gap area (NGAA)	Direct visualisation and still images from videolaryngoscopy	Yes	Kruskal-Wallis
Smalle et al. (2017) [15]	*Veterinary anaesthesia and analgesia*	Prospective randomized crossover, blinded	6 research dogs	Healthy	2006/6/6	No	Visual subjective	Subjective laryngeal exposure score	Yes	Friedman/Mann-Whitney U tests, Spearman
McKeirnan et al. (2014) [16]	*Journal of the American Animal Hospital Association*	Prospective randomized, blinded	48 shelter dogs	Healthy	24/24	No	Visual subjective	Subjective laryngeal exposure score	Yes	T test and Fischer exact test
Jackson et al. (2004) [6]	*Veterinary surgery*	Prospective randomized crossover	6 dogs	Healthy	6/6/6/6/6/6/6	No	Normalised glottal gap area (NGAA)	Video and still images from videolaryngoscopy	Yes	ANOVA/Students t test
		blinded								
Tobias et al. (2004) [17]	*Veterinary anaesthesia and analgesia*	Prospective experimental and clinical	12 dogs	Healthy/laryngeal paralysis	6-6	No	Normalised glottal gap area (NGAA)	Video and still images from videolaryngoscopy	Yes	Wilcoxon rank sum test/t-test/Mann-Whitney test
Gross et al. (2002) [18]	*Journal of the American Animal Hospital Association*	Prospective randomized crossover	8 dogs	Healthy	2008/8/8	No	Visual subjective	Direct visualisation	Yes	ANOVA
		blinded								
Miller et al. (2002) [19]	*Journal of Veterinary Internal Medicine*	Prospective	30 research dogs	Healthy	30	No	Normalised rima glottides surface area (RGSA)	Video and still images from videolaryngoscopy	Yes	Kolmogorov-Smirnov
										/ANOVA

**Table 2:** Characteristics and study design features from 8 studies evaluating the effects of premedication on laryngeal motion in dogs.

Premedication Agent	Dose	Induction Agent	Timing before Induction	Improved Examination Conditions	Results	Statistical Significance	Reference
Dexmedetomidine vs. Butorphanol + dexmedetomidine vs. Hydromorphone + dexmedetomidine	15 µg kg^−1^ IV dexmedetomidine 0.3 mg kg^−1^ IV butorphanol + 7 µg kg^−1^ IV dexmedetomidine 0.1 mg kg^−1^ IV hydromorphone + 5 µg kg^−1^ IV dexmedetomidine	No	To effect	Yes	Normal laryngeal motion with all protocols	No	DeGroot et al. (2019) [7]
Acepromazine + methadone	0.01 mg kg^−1^ IM acepromazine + 0.2 mg kg^−1^ IM methadone	Yes–alfaxalone/propofol	30 min prior to induction	N/D	>75% maintained laryngeal motion	N/D	Norgate et al. (2018) [12]
Acepromazine + butorphanol vs. saline (control)	0.03 mg kg^−1^ IV acepromazine + 0.2 mg kg^−1^ IV butorphanol vs. saline (non-premedicated control group)	Yes–alfaxalone/propofol	5 min prior to induction	Yes	No arytenoid motion in 50% of dogs	Yes	Radkey et al. (2018) [13]
Butorphanol	0.5 mg kg^−1^ IV butorphanol	Yes–propofol/ketamine	20 min prior to induction	N/D	N/D	N/A	McKeirnan et al. (2014) [16]
				good conditions			
Butorphanol	0.5 mg kg^−1^ IV butorphanol	Yes–thiopental/propofol	5 min prior to induction	N/D	Laryngeal motion observable	N/A	Gross et al. (2002) [18]
Acepromazine + butorphanol	0.2 mg kg^−1^ IM acepromazine + 0.4 mg kg^−1^ IV butorphanol	Yes–mask isoflurane	20 min prior to induction	N/D	Arytenoid motion maintained	Yes	Jackson et al. (2004) [6]
Acepromazine	0.05 mg kg^−1^ IM acepromazine	Yes–thiopental	20 min prior to induction	N/D	Arytenoid motion less than with thiopental alone	Yes	Jackson et al. (2004) [6]
Acepromazine + oxymorphone	0.05 mg kg^−1^ IM acepromazine + 0.05 mg kg^−1^ IV oxymorphone	No	20 min prior to induction	N/D	N/D	N/A	Jackson et al. (2004) [6]
Acepromazine + butorphanol	0.022–0.2 mg kg−^1^ IM acepromazine + 0.44 mg kg^−1^ IM butorphanol	Yes–mask isoflurane	20 min prior to induction	N/D	Laryngeal motion present in all healthy dogs but no motion in dogs with laryngeal paralysis	N/D	Tobias et al. (2004) [17]
Acepromazine + butorphanol	0.05 mg kg^−1^ SQ acepromazine + 0.22 mg kg^−1^ IV butorphanol	Yes–propofol	20 min/5 min prior to induction	N/D	N/D	N/A	Miller et al. (2002) [19]

**Table 3:** Results and study features design from 12 studies evaluating the laryngeal function after the administration of induction agents.

Induction Agent	Dose	Sedation	Titration of Induction	Examination Conditions/Exposure	Results	Statistical Significance	Reference
Propofol vs. methohexital vs. saline	6.8 mg kg^−1^ IV propofol 7.4 mg kg^−1^ IV methohexital Control IV (saline control group)	No	To effect	No differences	No differences in laryngeal motion among groups	No	Brown et al. (2019) [10]
Alfaxalone + doxapram vs. propofol + doxapram	1.5 mg kg^−1^ IV alfaxalone 3.0 mg kg^−1^ IV propofol 2.5 mg kg^−1^ IV doxapram	No	To effect	No differences	Alfaxalone-doxapram significantly less arytenoid motions	Yes	Labuscagne et al. (2019) [11]
Propofol vs. dexmedetomidine	6.5 mg kg^−1^ IV propofol 15 µg kg^−1^ IV dexmedetomidine	No	To effect	Good except one dog in propofol group	Laryngeal function observed in all except propofol	Yes	DeGroot et al. (2019) [7]
Alfaxalone vs. propofol + diazepamvs. thiopental	2.6 mg kg^−1^ IV alfaxalone 3.8 mg kg^−1^ IV propofol + 0.4 mg kg^−1^ IV diazepam 14.2 mg kg^−1^ IV thiopental	No	To effect	N/D	No differences in laryngeal motion among groups	No	Ambros et al. (2018) [14]
Isoflurane	3–5% in oxygen (2 L/min)	Yes	To effect	N/D	Active laryngeal motion detected in all healthy dogs but in none of the dogs with suspected laryngeal paralysis	N/A	Tobias et al. (2004) [17]
Thiopental vs. propofol vs. ketamine + diazepam vs. acepromazine + thiopental vs. acepromazine + propofol vs. isoflurane	14 mg kg^−1^ IV thiopental 5.6 mg kg^−1^ IV propofol 8.5 mg kg^−1^ IV ketamine + 0.4 mg kg^−1^ IV diazepam 0.05 mg kg^−1^ IM acepromazine + 9.8 mg kg^−1^ IV thiopental 0.05 mg kg^−1^ IM acepromazine + 3.7 mg kg^−1^ IV propofol N/D	Yes	To effect	N/D	After induction: no differences in laryngeal motion among groups. Prior to recovery, thiopental superior motion	No/Yes	Jackson et al. (2004) [6]
Propofol vs. thiopental vs. ketamine + diazepam	3.6 mg kg^−1^ IV propofol 10.4 mg kg^−1^ IV thiopental5.6 mg kg^−1^ IV ketamine + 0.3 mg kg^−1^ IV diazepam	Yes	To effect	Exposure lower in ketamine/ diazepam	Laryngeal function observed with all protocols.	No	Gross et al. (2002) [18]
Propofol	4.0 mg kg^−1^ IV propofol	Yes	No	N/D	N/D	N/A	Miller et al. (2002) [19]

**Table 4:** The effects of doxapram on the laryngeal motion in healthy dogs and dogs with laryngeal paralysis.

Respiratory Stimulant	Dose (bolus) /(Titration)	Health Status	Pre-medication	Induction of Anaesthesia	Adverse Effects	Results	Passive or Paradoxical Arytenoid Motion	Statistical Significance	Reference
Doxapram vs. control	2.2 mg kg^−1^ IV/saline (control)	Healthy	No	Propofol/methohexital	Exaggerated laryngeal movements	Doxapram improved breathing scores but not laryngeal function	No	No	Brown et al. (2019) [10]
Doxapram	1.0 mg kg^−1^ IV	Healthy	Dexmedetomidine/Butorphanol/Hydromorphone	Propofol/ dex-medetomidine	No	Doxapram improved laryngeal function in dogs receiving dexmedetomidine. No improvements in the other drug protocols	Yes, prior to doxapram in propofol group	Yes	DeGroot et al. (2019) [7]
Doxapram vs. Mechanical stimulation	2.5 mg kg^−1^ IV	Healthy	No	Alfaxalone/propofol/thiopental	No	Doxapram more effective in stimulating laryngeal motion. Examination time longest with alfaxalone, despite doxapram	No	Yes	Labuscagne et al. (2019) [11]
Doxapram	0.25 mg kg^−1^ IV	Healthy	Acepromazine + Butorphanol/ control group	Alfaxalone/propofol	Increased respiratory drive	After doxapram, laryngeal motion present in all healthy dogs with previously lacking laryngeal motion. RGSA was significantly less in ALF before doxapram compared with all other treatments and after doxapram 50% of dogs in alfaxalone no motion	Yes, in dogs with previously good motion	Yes	Radkey et al. (2018) [13]
Doxapram	1 mg kg^−1^ IV	Healthy	Butorphanol	Propofol/ketamine/propofol	None	Doxapram improved respiratory scores and significantly increased the ability to determine normal laryngeal function	No	Yes	McKeirnan et al. (2014) [16]
Doxapram	2–5 mg kg^−1^ IV	Healthy	Acepromazine /Butorphanol	Multiple	N/D	N/D	N/D	N/A	Jackson et al. (2004) [6]
Doxapram	1.1 mg kg^−1^ IV	Healthy and with laryngeal paralysis	Butorphanol/Acepromazine	Isoflurane by mask	Intubation necessary	Healthy dogs differentiated from dogs with laryngeal paralysis with doxapram	Yes, in dogs with laryngeal paralysis	Yes	Tobias et al (2004) [17]
Doxapram	2.2 mg kg^−1^	Healthy	Acepromazine + Butorphanol	Propofol	Excitement/awakening	Doxapram increased laryngeal motion in healthy premedicated dogs	No	Yes	Miller et al. (2002) [19]

**Table 5:** Quality assessment of the 12 studies included using the modified SIGN Criteria for RCTs and their assigned levels of evidence (LoE).

Quality Criterion.		Score	Labuscagne et al. (2019) [11]	Brown et al. (2019) [10]	DeGroot et al. (2019) [7]	Norgate et al. (2018) [12]	Radkey et al. (2018) [13]	Smalle et al. (2017) [15]	Ambros et al. (2018) [14]	McKeirnan et al. (2014) [16]	Tobias et al. (2004) [17]	Jackson et al. (2004) [6]	Gross et al. (2002) [18]	Miller et al. (2002) [19]
Clear question addressed by study	Yes	1	1	1	1	1	1	1	1	1	1	1	1	1
	No	0	/	/	/	/	/	/	/	/	/	/	/	/
Acceptable randomization method	Yes	1	1	1	/	1	1	1	/	1	/	1	/	/
	No	0	/	/	/	/	/	/	/	/	/	/	/	/
	N/R	0	/	/	0	/	/	/	0	/	0	/	0	0
Adequate concealment method	Yes	1	1	1	1	/	/	1	/	1	/	/	/	/
	No	0	/	/	/	/	/	/	/	/	0	/	/	/
	N/R	0	/	/	/	0	0	/	0	/	/	0	0	0
Blinding of assessors	Yes	1	1	1	1	1	1	1	1	1	/	1	1	/
	No	0	/	/	/	/	/	/	/	/	/	/	/	/
	N/R	0	/	/	/	/	/	/	/	/	0	/	/	0
Assessment videolaryngoscopy and direct observation	Yes	2	/	2	/	2	/	/	2	/	/	/	/	/
	No	0	/	/	/	/	/	/	/	/	/	/	/	/
	One only	1	1	/	1	/	1	1	/	1	1	1	1	1
Agreement between assessors	Yes	1	/	/	/	1	/	/	/	/	/	/	/	/
	No	0	/	0	/	/	/	/	/	0	/	/	/	/
	N/R or N/A	0	0	/	0	/	0	0	0	/	0	0	0	0
Groups similar at baseline	Yes	1	1	1	1	1	1	1	1	1	1	1	1	1
	No	0	/	/	/	/	/	/	/	/	/	/	/	/
Only difference between groups is the anaesthetic drug or doxapram	Yes	1	1	1	1	1	1	1	1	1	1	1	1	1
	No	0	/	/	/	/	/	/	/	/	/	/	/	/
Outcomes measurements are standard, valid and reliable	Yes	1	1	1	1	1	1	1	1	1	1	1	1	1
	No	0	/	/	/	/	/	/	/	/	/	/	/	/
Intention-to-treat (ITT)	Yes	1	1	1	1	1	1	1	1	1	1	1	1	1
	No	0	/	/	/	/	/	/	/	/	/	/	/	/
													
Overall bias rating	(++)													
	(+)		(+)	(++)	(+)	(++)	(+)	(+)	(+)	(+)	(−)	(+)	(+)	(+)
	(−)													
Level of evidence (LoE)	I-V		II	II	II	II	II	II	II	II	III	II	II	III

RCTS: Randomised controlled trials; N/R, not reported; N/A, not available. Level of evidence (according to Oxford Centre of Evidence-based Medicine 2011) (range 1 = highest to range 5 = lowest). SIGN=Methodology Checklist 2 of Controlled Trials: Low risk of bias (++), medium risk of bias (+), high risk of bias (−).
